# Temporary Sternoclavicular Plating for an Unusual Double Clavicle Fracture (Medial Nonunion, Lateral Acute) Complicated by an Intraoperative Pneumothorax

**DOI:** 10.1155/2014/206125

**Published:** 2014-09-02

**Authors:** John G. Skedros, Alex N. Knight, Chad S. Mears, Tanner D. Langston

**Affiliations:** ^1^Department of Orthopaedic Surgery, The University of Utah, Salt Lake City, Utah, USA; ^2^Intermountain Medical Center, Salt Lake City, Utah, USA; ^3^Utah Orthopaedic Specialists, Salt Lake City, Utah, UT, USA

## Abstract

Double (segmental) clavicle fractures, involving both the medial and lateral aspects of the clavicle, are very uncommon. Even less common is an asynchronous double fracture with one of the fractures being a nonunion. We report the case of a 30-year-old healthy male patient who had an unusual double clavicle fracture (medial nonunion, lateral acute) that occurred in separate traumatic events during motocross (motorcycle) racing. His fractures were treated surgically in two stages. In the first stage a long reconstruction plate was used that spanned onto the sternum and two transcortical screws were placed into the manubrium to enhance purchase for the deficient bone of the medial clavicle. In accordance with the preoperative plan, the medial one-third of the plate and the medial four screws (of the total 13 used) were removed. Although our patient had an excellent final result, he did have an intraoperative pneumothorax that was treated uneventfully with a chest tube. Medial clavicle fractures are difficult to treat, especially if they are nonunions and surgical complication rates can be high. Our case is one of the few that has been described where temporary sternoclavicular plating was successful in achieving an excellent long-term outcome.

## 1. Introduction

Fractures of the clavicle account for 2–5% of all fractures and approximately 35% of injuries to the shoulder girdle [1–3]. Nearly one-fourth of clavicle fractures occur at the lateral end [4–6] and those that occur at the medial end are usually (~85%) the result of motor vehicle trauma [7]. For example, Throckmorton and Kuhn [7] described 57 medial clavicle fractures in 55 patients. Operative treatment was rarely performed and the long-term outcome was usually good. However, 20% of the patients died within one month of their injuries, which reflects the multisystem trauma that is often associated with these fractures. Although the majority of medial clavicle fractures can be treated satisfactorily by nonoperative means, about 8% become symptomatic nonunions especially when they are displaced [1, 8]. Surgical treatment of medial clavicle nonunions is also associated with high complication and failure rates, particularly hardware migration [8–13].

Double (segmental) clavicle fractures, involving both the medial and lateral aspects of the clavicle, are rare. Furthermore, pneumothorax associated with a clavicle fracture is uncommon, occurring in approximately 3% of cases [14, 15]. We report a case with several unusual aspects, including (1) the patient had an asynchronous double clavicle fracture that included a symptomatic fracture nonunion at the far medial aspect of the shaft and an acute fracture at the lateral shaft, (2) the preoperative plan was to temporarily span the plate-and-screw fixation medially onto the sternum in order to rigidly stabilize the medial reconstruction and then remove the medial portion of the plate three months later, and (3) the index procedure was complicated by an intraoperative pneumothorax.

## 2. Case Report 

**Figure 1:**
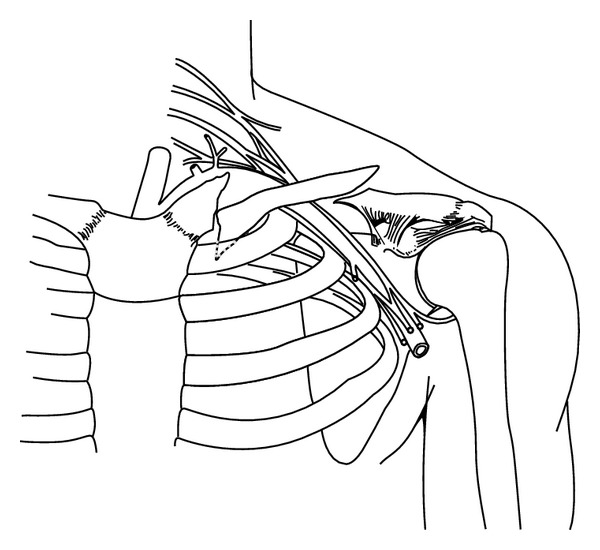
Drawing based on the first post-injury radiograph taken of the patient's left clavicle. This shows the double fracture pattern: a medial nonunion with an inferiorly directed spike of bone and an acute lateral fracture. The inferiorly directed spike of the medial fracture was in close proximity to the lung apex. The actual radiograph could not be used to provide this illustration because of water damage.

**Figure 2:**
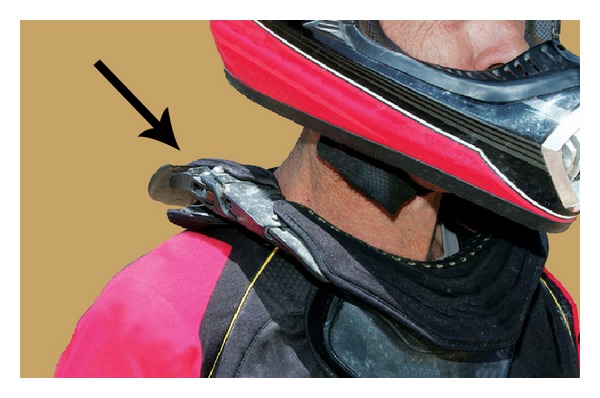
A motocross rider (not our patient) shown wearing a Leatt neck brace (arrow).

The patient is a 33-year-old healthy right-hand-dominant male who worked as an electrician and presented to our clinic after sustaining a closed left clavicle fracture in a motocross (motorcycle) race accident. The acute fracture was at the lateral third of the clavicle shaft and there was an established fracture nonunion at the medial shaft (type 1B1) [13] (Figure 1). At the time of this recent injury he was wearing a Leatt brace, which is designed to decelerate the head and protect the cervical spine during a crash (Figure 2). There was no neck or chest wall injury, loss of consciousness, or other significant injuries. He did not seek medical attention until appearing in our clinic one week later.

The patient reported having a history of “several left clavicle fractures”, the first occurring in high school in a motocross accident. He received little medical attention and only one radiograph was taken. Three years later in another motocross accident he stated that he “fractured the same clavicle in the same place as before”. In view of the persistent pain with crepitus it is likely that the fracture never healed. With respect to the biomechanics of the chronic medial fracture, a direct blow from striking the handle bars of the motorcycle initially caused this fracture. By contrast the lateral fracture was from indirect trauma, with the fracture occurring at the outer edge of the Leatt brace.

No surgeries had been performed on the clavicle. However, over the two years prior to being seen in our clinic the medial clavicle nonunion had become increasingly painful with overhead activities, which reflected the greater demands of his work as an electrician. In the setting of the acute lateral-shaft fracture, the patient requested surgery to address both the nonunion and the acute fracture.

**Figure 3:**
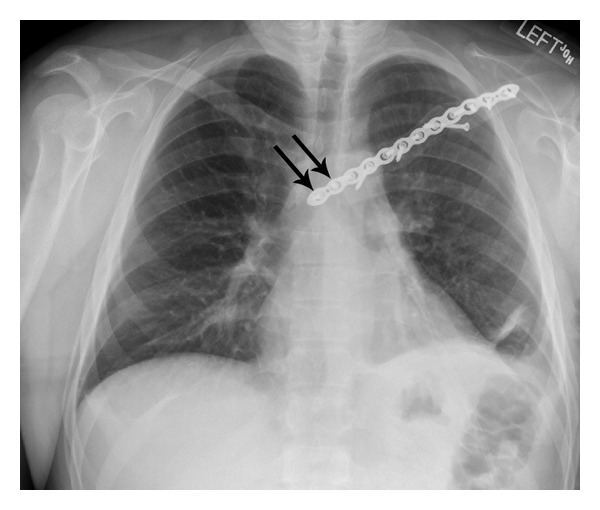
Fixation of both fractures using a reconstruction plate with screws. The arrows indicate the two screws placed in the sternum.

Both fractures were treated with open reduction with internal fixation (ORIF) using a 12-hole nonlocking reconstruction plate and thirteen 3.5 mm screws. In accordance with the preoperative plan, the medial scapula nonunion was treated with realignment, autogenous bone graft, and ORIF; the latter included spanning the plate onto the sternum in order to provide rigid fixation for the medial reconstruction (Figure 3). This was done because it was deemed necessary to place four screws, purchasing eight cortices, medial to the medial fracture (two in the medial clavicle and two in the sternum). The rationale for doing this was (1) the reported difficulty in obtaining enough screw purchase into the small medial clavicle fragment [12, 16–18] and (2) our initial impression that our patient might be noncompliant with lifting and shoulder motion restrictions, which could lead to nonunion if rigid fixation was not employed. Also, the only other reports that we could locate at that time that described sternoclavicular plating (for fracture or sternoclavicular dislocation) used a hook plate [16, 19], which is designed for a lateral clavicle fracture [20]. We were concerned a hook plate would not provide sufficient rigidity for our patient.

**Figure 4:**
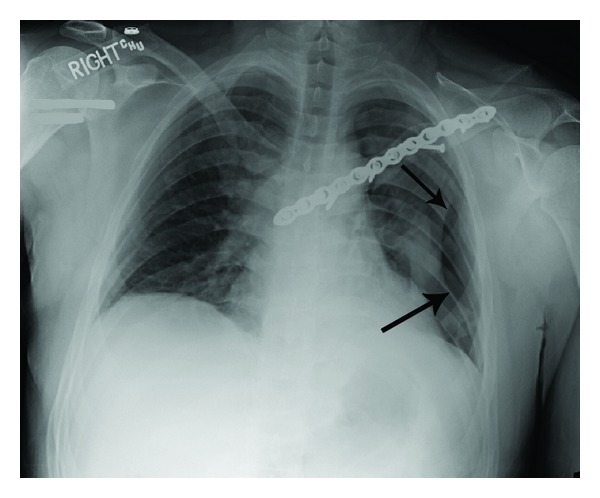
Chest radiograph taken in the recovery room after surgery. The arrows indicate the pneumothorax.

The surgery was done using a beach chair position and an X-ray C-arm intensifier to ensure accurate screw placement. Although the surgery seemed uneventful, the patient had shortness of breath in the recovery room and a pneumothorax was diagnosed with an anterior-posterior (AP) radiograph (Figure 4). The pneumothorax was not detected intraoperatively with the C-arm X-ray intensifier because only the upper lung could be seen clearly, the remainder being obscured by the operation table. A chest tube was placed in the recovery room. The chest tube was removed without complications two days later.

**Figure 5:**
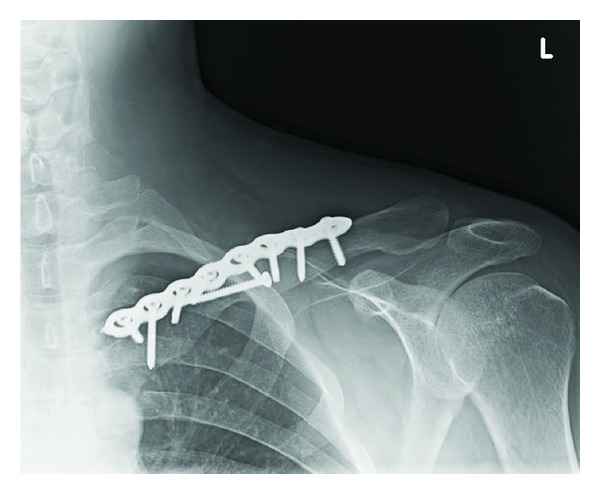
Radiographs taken after removing the medial one-third of the plate and four screws.

Three months later, the medial one-third of the plate was cut and it was removed along with the four medial screws as planned (Figure 5). Preoperative radiographs showed that the medial fracture was healing well. The remainder of the plate and the remaining screws had not been removed at final follow-up 3.5 years later. The patient reported complete satisfaction with his final result, which included no pain or dysfunction and had a final DASH (disability of arm, shoulder, and hand) score of 8 (0 = best, 100 = worst) [21]. He had also returned to full work capacity by five months after the index surgery.

## 3. Discussion

Reconstruction of medial clavicle fractures can be difficult because of the limited bone available medially for adequate fixation. It is for this reason that various fixation devices have been used for medial clavicle fracture reconstruction. For example, pins, wires, lag screws, small fragment reconstruction plates, dynamic compression plates, and other metal plates adapted or modified for this anatomical site have been described and usually with success [12, 13, 16, 18, 22]. Figure-of-eight sutures have also been used to stabilize a medial clavicle fracture in a 14-year-old boy [23]. Kim et al. [17] reported successful surgical treatment of a displaced medial clavicle fracture using a small T-shaped plate and multiple tension band sutures. Plate and screw fixation seems to be safer than pin or wire fixation [24]. However, medial clavicle fracture nonunion due to implant migration has been reported in a patient treated with a small fragment reconstruction plate [12].

To improve the strength of fixation of medial clavicle fractures, Gille et al. [16] employed a novel technique of fixing a reconstruction device into the sternum. They treated an acute medial clavicle fracture with the temporary implantation of a hook plate designed for lateral clavicle fractures. They modified the plate so that it could be inserted into a hole made in the sternum. In accordance with their preoperative plan, the plate was removed three months later and the fracture healed without complication. We advanced this concept by applying two transsternal screws, which supplemented the two screws that were placed through the medial most portion of the clavicle.

In a study published after our patient had his index surgery, Oe et al. [13] reported the results of 10 operatively treated medial clavicle fractures with locking or nonlocking standard (longitudinal) plates, T-shaped plates, or pilon reconstruction plates. They recommended the use of at least three screws in the medial bone for rigid fixation. In two cases, the plates (pilon type) spanned the sternoclavicular joint. The other eight patients had hardware that did not cross the sternoclavicular joint. They recommended removing the hardware at 18 months, but the actual removal times were 4, 9, 11, 18, 19, 19, and 32 months (two patients without removal). The four-month removal time was for one of the patients with sternoclavicular plating. But the other patient with the sternoclavicular plating developed a nonunion, which ultimately required removal of two-thirds of the clavicle, with a poor result (final DASH score: 66.7; 0 = best, 100 = worst). The DASH score for the other patients was typically good at final follow-up (range: 0–16).

As illustrated by some of the above mentioned reports, fixation of medial clavicle fractures that cross the sternoclavicular joint can have very significant complications that would restrict its use to only a small percentage of cases. Complications might also include (1) drill bit or screw penetration into the pericardial tissues or deeper, (2) sternum fracture during unadvised postoperative use, and (3) nonunion [10, 11, 13, 25–27]. We carefully weighed these risks and our patient was also given a detailed informed consent form in addition to an educational session that included a demonstration using a model of the shoulder girdle and thorax in order to describe potential complications. Fortunately our patient's fracture healed without hardware or healing complications.

Another concern with sternoclavicular plating as a treatment for medial clavicle fractures (acute and nonunions) is the motion that naturally occurs there with functional use of the shoulder [28, 29]. This could be a potential source of implant loosening even if the patient is relatively compliant. In other words, even rather minor activities of daily living (e.g., dressing and grooming) might result in sternoclavicular motion that could compromise the rigidity of the fracture reconstruction. To avoid this potential problem, Al-Yassari et al. [9] “offloaded” the sternoclavicular joint in their reconstructions for medial clavicle fractures or sternoclavicular joint instability. They accomplished this with a two-stage surgery where the first stage included fixation of the medial clavicle (which did not span the sternoclavicular joint) and a midclavicle osteotomy to “offload the fixation”. The second stage, at an average of four months after the index surgery, was the removal of the medial clavicle fixation and application of plate and screw fixation of the midclavicle osteotomy. From 1997 to 2007, they treated nine consecutive patients with an average age of 35 years: six patients had sternoclavicular joint instability and three had symptomatic medial clavicle fracture nonunion. The average follow-up was 41 months (range: 7–127). Although this method avoids sternoclavicular plating and in theory enhances the healing of the clavicle fracture by reducing untoward motion, it does potentially require three surgeries—the final one being removal of the plate used in the second stage. For this reason, sternoclavicular plating seems to be a more attractive option because (1) it is likely that all of the hardware could be removed without complication by four months postoperatively, and (2) no adverse events with respect to implant migration were reported by Franck et al. [19] and Gille et al. [16]. Additional studies are needed in order to determine if sternoclavicular motion that naturally occurs (even in patients who are compliant with motion and lifting restrictions) during the first four postoperative months could compromise rigid sternoclavicular plating before healing is well established. Locking plates could also be used to enhance the rigidity of the sternoclavicular fixation [13].

The perceived benefits of sternoclavicular plating for fixation of* acute* medial clavicle fractures would also have to exceed the uniformly favorable results reported by Low et al. [18]. These investigators performed open reduction and internal fixation on displaced, medial clavicle fractures in five adult patients (aged 25–52 years, mean 43; including one patient with a nonunion). The fixation included a medial clavicle plate and screws in four cases and stabilized in another with only one screw and sutures because of poor bone stock. No hardware was placed into the sternum or across the sternoclavicular joint. The mean follow-up was 3.3 years (range: 8 months—10 years). All fractures united clinically and radiographically, and there were no complications and no revision surgery required. All patients had full range of motion of their shoulder at final follow-up and were able to return to preinjury occupational and activity levels. However, in contrast to these good results, the fixation of medial clavicle fracture* nonunions* has a higher rate of unsatisfactory results, which favors the consideration for sternoclavicular plating [12, 13, 30].

One of the unusual and concerning aspects of our case was the pneumothorax that occurred during surgery. This complication was not a result of the sternal plating; rather it occurred during the dissection of the most inferior aspect of the fracture fragment at the nonunion site. We advise surgeons to be aware of this problem when dissection is near the lung apex. Intraoperative chest radiographs are recommended if there is any suspicion that this complication occurred. However, it is also possible that an intraoperative chest radiograph could miss an intraoperative pneumothorax for a variety of reasons, which may warrant repeated plane radiographic imaging or computed tomography of the chest after surgery [31].

## 4. Conclusion 

Our patient had an unusual double clavicle fracture (medial nonunion, acute lateral) that was treated with a two-stage procedure. In the first stage a long reconstruction plate was used that spanned onto the sternum with two screws. Three months later the medial one-third of the plate and screws were removed. Although our patient had an excellent final result, he did have an intraoperative pneumothorax that was treated uneventfully with a chest tube.
